# Homonuclear ^1^H NMR and circular dichroism study of the HIV-1 Tat Eli variant

**DOI:** 10.1186/1742-4690-5-83

**Published:** 2008-09-22

**Authors:** Jennifer D Watkins, Grant R Campbell, Hubert Halimi, Erwann P Loret

**Affiliations:** 1 Unité mixte de recherche Université de la Méditerranée/INSERM U911, Faculté de Pharmacie, Université de la Méditerranée, 27 Boulevard Jean Moulin, 13385 Marseille, France; 2 Department of Pediatrics, Division of Infectious Diseases, University of California San Diego, La Jolla, California, USA

## Abstract

**Background:**

The HIV-1 Tat protein is a promising target to develop AIDS therapies, particularly vaccines, due to its extracellular role that protects HIV-1-infected cells from the immune system. Tat exists in two different lengths, 86 or 87 residues and 99 or 101 residues, with the long form being predominant in clinical isolates. We report here a structural study of the 99 residue Tat Eli variant using 2D liquid-state NMR, molecular modeling and circular dichroism.

**Results:**

Tat Eli was obtained from solid-phase peptide synthesis and the purified protein was proven biologically active in a trans-activation assay. Circular dichroism spectra at different temperatures up to 70°C showed that Tat Eli is not a random coil at 20°C. Homonuclear ^1^H NMR spectra allowed us to identify 1639 NMR distance constraints out of which 264 were interresidual. Molecular modeling satisfying at least 1474 NMR constraints revealed the same folding for different model structures. The Tat Eli model has a core region composed of a part of the N-terminus including the highly conserved Trp 11. The extra residues in the Tat Eli C-terminus protrude from a groove between the basic region and the cysteine-rich region and are well exposed to the solvent.

**Conclusion:**

We show that active Tat variants share a similar folding pattern whatever their size, but mutations induce local structural changes.

## Background

The human immunodeficiency virus type 1 (HIV-1) trans-activator protein Tat is essential for the activation and expression of HIV genes [1]. Tat interacts with a RNA hairpin-loop structure called the trans-activation-responsive region (TAR) located at the 5' end of all nascent viral transcripts and interacts with an RNase suppressing the processing of small RNAs [2,3]. However, Tat differs from other HIV-1 regulatory proteins due to its early secretion from HIV-1-infected CD4^+ ^T cells [4]. Extracellular Tat can traverse cellular membranes and induce apoptosis preventing the immune system from eliminating HIV-1-infected cells [5]. Tat is encoded by two exons. The first exon encodes amino acids 1–72 and the second exon encodes amino acids 73–86/101 that contribute to viral infectivity and other functions such as the induction of CD4^+ ^T cell apoptosis [6].

A vaccine targeting Tat could help restore cellular immunity in HIV-1-infected patients [7]. A recent study using autologous dendritic cells, loaded with exogenous simian immunodeficiency virus peptides that spanned the overlapping reading frames within Tat successfully induced cellular immune responses in rhesus macaques [8]. However, no successful phase II clinical trial targeting Tat has so far been reported [9]. This might be due to the variability of Tat variants, as Tat can tolerate up to 38% sequence variation that modifies its immunological epitopes without a loss in trans-activational activity [10]. Moreover, until now, most Tat vaccine approaches have used the European Tat Bru or HXB2 variant that have 86 residues [11], while Tat variants found in clinical isolates are predominantly 99 to 101 residues in length and have greater trans-activational activity [2,6,12].

All Tat variants with proven biological activity display similar circular dichroism (CD) spectra, while inactivation due to chemical cysteine modification dramatically changes the CD spectrum of Tat [12]. Tat is usually divided into six different regions [13]: region I (residues 1–21) is a proline-rich region and has a conserved Trp 11, region II (residues 22–37) has seven conserved cysteines at positions 22, 25, 27, 30, 31, 34 and 37 (no other cysteines are found in the sequence), region III (residues 38–48) has a conserved Phe 38 and the conserved sequence LGISYG from residues 43 to 48, region IV (residues 49–59) is rich in basic residues and has the conserved sequence RKKRRQRRRPP, region V (residues 60–72) is a glutamine-rich region, and region VI constitutes the C-terminus of Tat encoded by the second exon, but its size depends on the HIV-1 isolates. The nuclear magnetic resonance (NMR) structure of two active Tat variants of 86 and 87 residues (Tat Bru and Tat Mal respectively) showed a similar folding, while amino acid sequence variation led to local structural dissimilarities notably in region V [14,15]. A part of region I involving the strictly conserved Trp 11 constituted the core region, with the other regions packing around it while being well exposed to solvent. Recently, an NMR study of a peptide corresponding to the first exon of Tat (residues 1–72) showed that no structure could be identified in this peptide [16].

In this study, we report a complete NMR assignment and structural characterization of a long Tat variant (99 residues) called Tat Eli. HIV-1 Eli is a subtype D primary isolate identified during the 1980's in what was then Zaire [17]. Tat Eli was obtained from solid-phase peptide synthesis and has biological activity as demonstrated in a trans-activation assay. Circular dichroism (CD) experiments indicate that Tat Eli is not a random coil at 20°C. 2D NMR spectra of Tat Eli and molecular modeling revealed a folding similar to Tat Bru and Tat Mal for the first 86 residues. The C-terminal extension is exposed to solvent and is packed between the basic region and the cysteine-rich region.

## Results

### Synthesis and biological activity of Tat Eli

**Figure 1:**
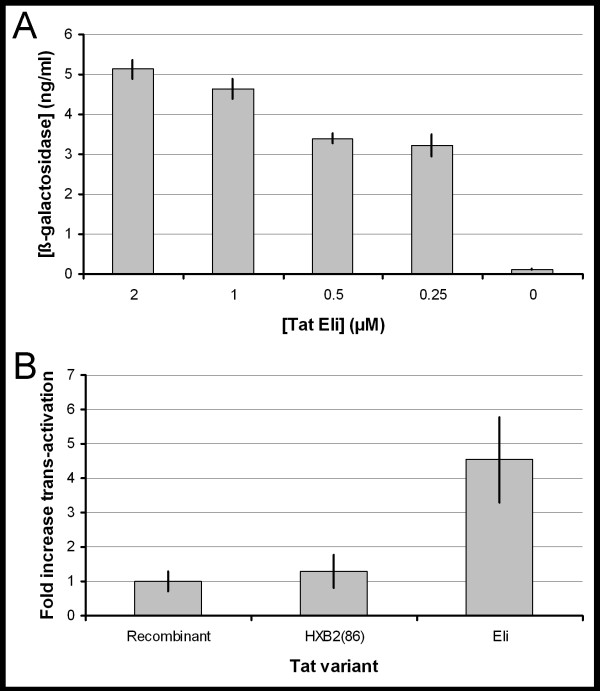
**Trans-activation assay with HeLa cells transfected with a HIV-1 LTR *lacZ *construct**. (A) The histograms show the trans-activation observed with synthetic Tat Eli using four different concentrations: 2 μM, 1 μM, 0.5 μM, and 0.25 μM. Without Tat, there is a basal expression of β-galactosidase as indicated with *control*. Error bars represent the standard deviation measured between two independent experiments carried out in triplicate. (B) The histograms show the fold difference in trans-activational activity observed with synthetic Tat Eli and synthetic Tat HXB2(86) compared with recombinant Tat at 50 nM, with recombinant Tat activity set at 1. Error bars represent the standard deviation measured between two independent experiments carried out in triplicate.

The chemical synthesis of Tat Eli was performed in a single run using Fast Fmoc chemistry. The synthesized protein had 99 residues and a molecular mass of 11081 (data not shown). Amino acid analysis revealed an amino acid content compatible with Tat Eli, and sequencing of the first five residues from the N-terminus gave a sequence identical to Tat Eli (data not shown). A trans-activation assay was performed and showed that the synthetic protein had trans-activational activity (Figure 1A). This assay closely resembles the natural conditions for extracellular Tat as the synthetic protein was added to the culture, and had to cross the cell membranes before binding to the nucleotide target TAR, triggering trans-activation. We compared the trans-activational activity of this synthetic Tat Eli with both a synthetic subtype B Tat (HXB2(86)) and with a recombinant subtype B Tat. We show that our synthetic B Tat had the same trans-activational activity as the recombinant subtype B Tat, but that Tat Eli had 4.5 fold more activity at the same concentration tested (Figure 1B).

### CD Spectra of Tat Eli

**Figure 2:**
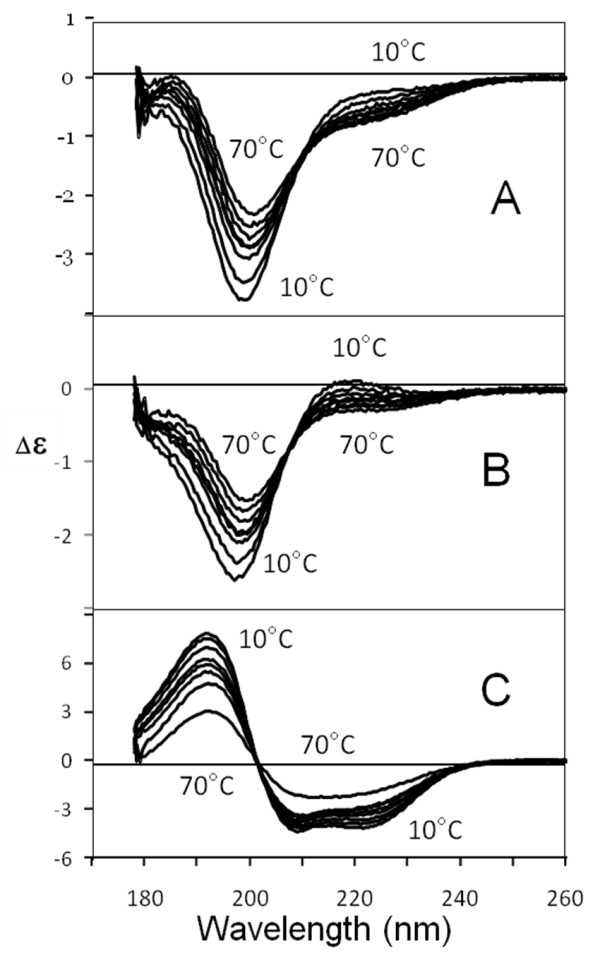
**Circular Dichroism of Tat Eli and control proteins at different temperatures**. CD spectra of Tat Eli (A), Protamine (B), and BSA (C). CD spectra were measured from 260 to 178 nm at different temperatures (10, 20, 30, 37, 40, 50, 60, and 70°C) in 20 mM phosphate buffer pH 4.5. Protamine has mainly β-turns in its structure while α-helices are predominant in the structure of BSA. If Tat Eli was a random coil, CD spectra should have been similar at all temperatures tested. This is not the case as the Tat Eli CD signal decreases with the increase in temperature (A) as is the case for the two control proteins (B and C).

**Figure 3:**
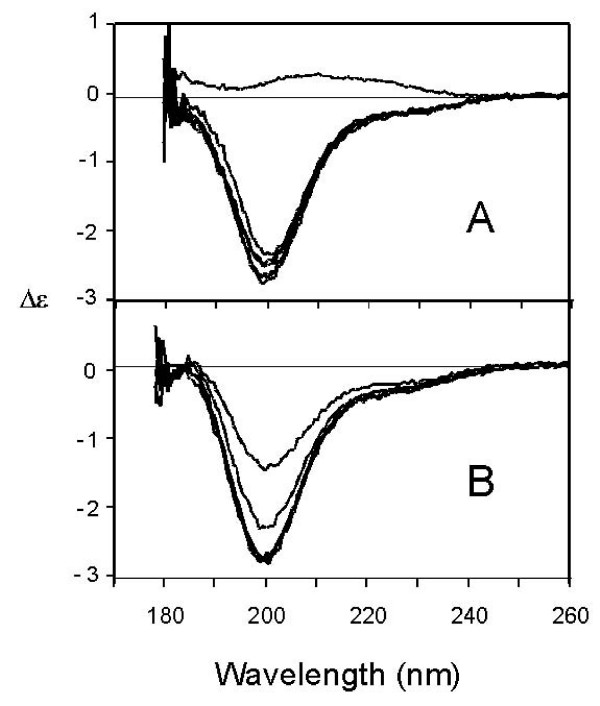
**Circular dichroism spectra of Tat Eli in the presence of Zn^2+^**. CD spectra of Tat Eli were measured from 260 to 178 nm at 20°C with increasing molar equivalents of Zn^2+ ^(0, 1, 2, 3, 4, 8 and 16) in 20 mM phosphate buffer pH 4.5 (panel A) and pH 7 (panel B). A full precipitation occurs with a Zn^2+ ^ratio 1:16 at pH 4.5 (A) while a precipitation starts with a ratio 1:8 at pH 7 (B). The binding of Zn^2+ ^does not modify the structure of Tat Eli for the ratio 1:1, 1:2, 1:3 and 1:4.

Tat Eli gives a CD spectrum with a main negative band close to 200 nm (Figure 2A). This is similar to the CD spectrum of a random coil peptide model [18]. However, a random coil-like CD spectrum is also observed in proteins such as protamine that have a stable structure and only β-turns as secondary structures [19]. Furthermore, as one is unable to differentiate between static and dynamic structures using CD, one cannot associate a CD band to random coils [18]. Therefore, to evaluate if Tat Eli could be a random coil we measured CD spectra over a range of temperatures under denaturing conditions. Under these conditions a random coil protein should display similar CD spectra at all temperatures tested. We also compared the CD spectra of Tat Eli at different temperatures with those of two other proteins: bovine serum albumin (BSA) and protamine (Figure 2). We observed a decrease in the CD signal intensity for all three proteins when the temperature was raised (Figure 2). According to CD theory, secondary structures have a CD specific signal due to a resonance phenomenon resulting from repetitive and similar Phi and Psi dihedral angles [18]. The melting of secondary structures should induce a decrease of CD signal as is illustrated in our experiments with the collapse of the α-helix signal of BSA (Figure 2C). The decrease in CD signal is not due to a reduction in solubility or aggregation, as the absorption spectra were similar at each temperature tested (data not shown). It is interesting to note that the CD signal of Tat Eli and protamine are almost similar at 70°C revealing that these two proteins have probably become random coils. The fact that Tat Eli has CD spectra markedly different at lower temperature indicates that Tat Eli is not a random coil at least at 20°C and CD data analysis (data not shown) reveals the presence of secondary structures such as extended structures (22%), β-turns (31%) and almost no α-helix (5%). Other structures with no repetitive dihedral angles represent 42% of the residues. We then tested the effect of Zn^2+ ^on Tat structure as previous reports stated that Tat binds Zn^2+ ^through its cysteine-rich region and that binding of Zn^2+ ^affects Tat CD spectrum and structure [20-22]. We tested different molar ratios of Tat Eli to Zn^2+ ^from 1:1 through 1:16 and the only effect observed was precipitation of Tat at 1:16 at pH 4.5 and 1:8 at pH 7 (Figure 3). When there is no precipitation, the CD spectra remain similar whatever the Tat:Zn^2+ ^ratio. Therefore, the binding of Zn^2+ ^does not modify the structure of Tat Eli.

### NMR Resonance Assignments

**Figure 4:**
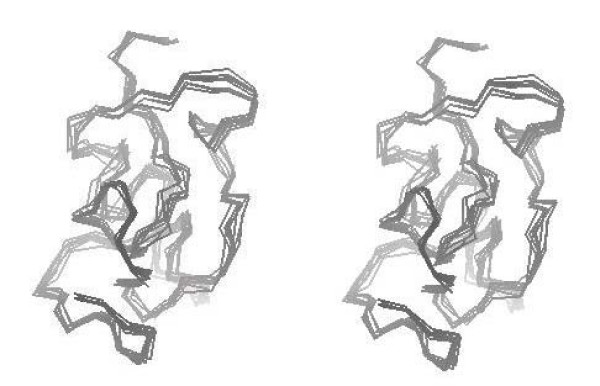
**Stereo view of the α-carbon chains of model structures of Tat Eli obtained from NMR constraints**. The structures were determined using simulated annealing and satisfied 1474 NMR distance constraints. A similar folding is observed with these different model structures.

The following spin systems were identified from the TOCSY spectrum: 28/28 Asp, Cys, His, Phe, Ser, Tyr and Trp; 14/14 Gln, Glu and Met; 9/9 Gly, 2/2 Ala, 16/16 Pro, 16/18 Arg and Lys, 2/2 Ile, 7/7 Val and Leu, and 3/3 Thr. The homonuclear ^1^H NMR spectra of Tat Eli allowed sequential assignment of all 99 spin systems by exploiting chemical shift similarity to previous 2D NMR assignments of the two short active Tat variants Tat Bru and Tat Mal [14,15]. Interestingly, the chemical shifts were similar to Tat Bru and Tat Mal [14,15]. No NOE-back calculation procedure was necessary to assign Tat Eli spin systems as was the case for Tat Bru [14]. The sequential assignment for these spin systems was obtained using the space connectivities H^α^(*i*)-H^N^(*i*+1), side chain H^N^(*i*+1) as H^β^*i*)-H^N^(*i*+1) and side chain H^α^(*i*). The unique spin systems corresponding to Trp 11, Phe 38, Ile 45, and Ile 69 were used as starting points and allowed the complete sequential assignment. The aromatic spin systems were identified from the ^1^H NOESY spectra using connectivities observed between the aromatic and the β and/or α protons. The ^1^H chemical shifts of Tat Eli are listed in Additional file 1. It was not possible to identify the H^N ^of Gln 72, Lys 89, and Lys 90 (Additional file 1). Although the H^α ^of the prolines have a low dispersion in the NOESY spectra, we were able to identify all of them using sequential H^δ^(*i*)-H^N^(*i*-1) and H^α^(*i*-1) connectivities. From the proton assignment, we identified 1639 NMR distance constraints out of which 264 were interresidual, 179 were sequential (*i*, *i*+1), 34 medium [(*i*-*j*) < 5], and 51 long range [(*i*-*j*) ≥ 5]. More than 15% of the long-range constraints involved the second exon of Tat, and half of those were in relation to the cysteine-rich region showing that the second exon is essential in Tat Eli folding (Figure 4).

### Conserved folding among Tat variants

**Figure 5:**
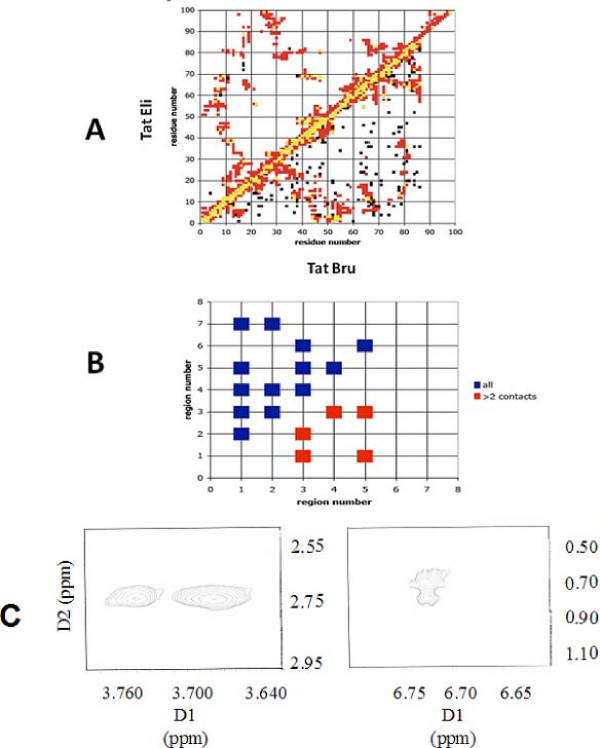
**Conserved folding among Tat variants**. (A) Conserved folding between Tat Bru (bottom) and Tat Eli (top): *Black *represents pair of residues with at least one experimental NMR constraints between them, *red *represents pairs of amino acids with a distance less than 5 Å in the calculated structures and *yellow *represents pairs of amino acids with both experimental NMR constraints and a calculated distance less than 5 Å. (B) Contacts between the different regions of Tat Eli: the figure shows the regions that have one or more contact(s) and the number of contacts between them including or excluding the i, i+1 contacts. Red symbols in the lower triangle show regions that have three or more inter-region NMRconstraints. (C) Contour plot showing connectivities between Hβ of Cys 25 and Hδ of Pro 99 and Hβ of Ser 93 and Hβ of Asp 98 (left panel) between the aromatic ring protons of Tyr 47 and Hδ of Leu 69 (right panel).

The sequence of Tat Eli is very similar to Tat Mal [15]. Therefore, we chose to compare NMR constraints of Tat Eli with Tat Bru that has 25% sequence variation with Tat Eli (Figure 5A) [14]. The two proteins have a similar folding despite the fact that there are less NMR constraints for Tat Eli due to its high flexibility. Figure 5B shows the contacts between the different regions of Tat. We can deduce that region III is more stable because it is interacting with regions I, II, IV and V. Interestingly, even if the beginning of region III is highly variable among Tat variants, the sequence between residues 42 and 51 is the most conserved. Moreover, NMR data allow us to confirm the presence of two β-turns (^9^EPWN and ^45^YSIG) although CD experiments indicate that Tat Eli has around 30% of β-turn secondary structures. This could be due to the superposition of spin systems on Tat Eli's spectra. Long distance NMR constraints were identified between the extra residues of the C-terminus and residues of regions II, III and V (Figure 5C).

### Tat Eli structure

**Figure 6:**
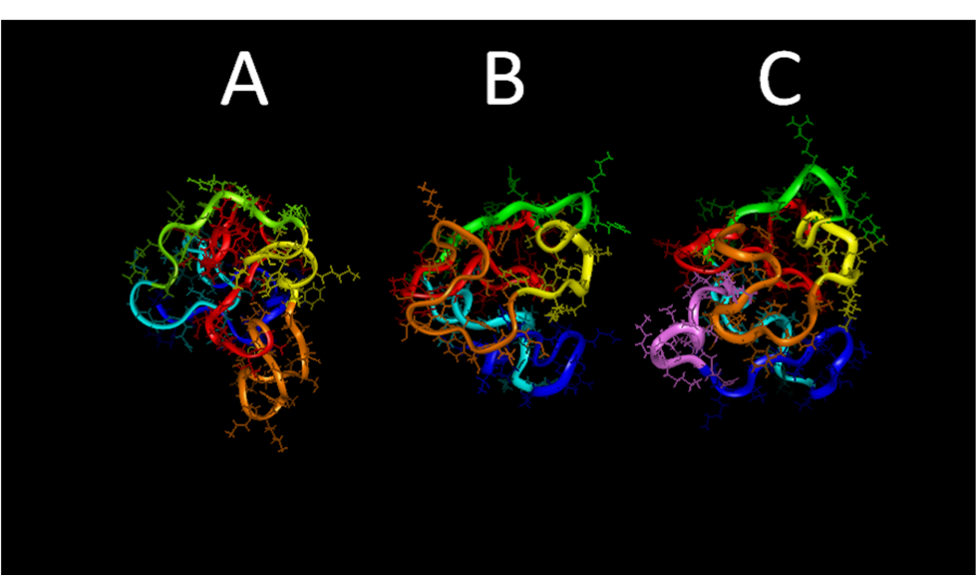
**Tat Bru (A), Tat Mal (B), and Tat Eli (C) 3D structures**. Region I is depicted in *red*, region II (cysteine-rich region) in *orange*, region III in *yellow*, region IV (basic region) in *green*, region V in *light blue*, region VI (residues 73–86/87) in *blue *and for Tat Eli the extra C terminal residues are in *pink*. The three Tat structures displayed a similar folding characterized by a core region composed of a part of region I with the highly conserved Trp 11 while the functional region II, IV and V are well exposed to the solvent. The extra residues in the C-terminus of Tat Eli protrude from a groove between the basic region and the cysteine-rich region and are exposed to solvent.

Model structures of Tat Eli were determined with NMR constraints using a simulated annealing protocol [23]. Superimposition of conformers with the lowest van der Waals energies, Coulombic energies, and respect of NMR constraints shows a similar folding (Figure 4). The mean structure was then refined by energy minimization without NMR constraints but with a freeze backbone (Additional file 2). Although we found specific NMR constraints for only two β-turns, model structures reveal that Tat Eli has eight β-turns in agreement with CD data. Figure 6C shows the structure of Tat Eli compared to Tat Bru and Mal (Figures 6A and 6B). In region I, we found two β-turns involving residues ^9^EPWN and ^17^QPRT as for Tat Mal [15]. The cysteine-rich region (region II) is constituted of two loops which are well exposed to solvent. Region III begins with a loop followed by a β-turn starting from Ile 45. This turn was also found in Tat Bru and Tat Mal, corresponding to a well-conserved sequence among Tat variants [14,15]. The basic region (region IV) adopts an extended structure similar to Tat Bru and Tat Mal. The glutamine-rich region (region V) is composed of two β-turns involving residue ^63^QAHQ and ^70^PKQP. The C-terminal region of Tat Eli (region VI) is composed of three β-turns involving residues ^76^QPRG as for Tat Mal, ^83^GPKE as for Tat Bru and ^91^VESE, not present in the shorter Tat variants. Furthermore, the core of Tat Eli is mainly composed of region I, with Trp 11 at a central position that is part of a hydrophobic cluster containing Phe 38 and Tyr 47. This is the same core as in both Tat Bru and Tat Mal [14,15].

## Discussion

This is the first NMR study of a long Tat variant (99 residues) with biological activity. CD data show that the synthetic Tat Eli used in our 2D NMR study is not a random coil. We observed similar chemical shifts with the two previous NMR studies of biologically active Tat variants [14,15] suggesting a common folding for Tat. This is characterized by a central location of the N-terminal region around the highly conserved Trp 11 that is part of a hydrophobic pocket that contains well-conserved aliphatic and aromatic residues.

Our results are different from the NMR study of a peptide corresponding to the first exon of Tat suggesting that Tat is a natively unfolded protein [16]. This study was remarkably well done from the point of view of NMR; however, it was carried out on a peptide that does not correspond to a real Tat protein. Moreover, the sequence used does not correspond to a primary isolate, as a viable HIV-1 strain that expresses only the first exon of Tat has never been observed, and it has been shown that both exons of Tat are necessary for integrated proviruses [24]. Furthermore, the second exon of Tat has important functions for replication *in vivo *[25] and is involved in CD4^+ ^T cell apoptosis [6]. We were able to identify long-range NMR constraints with our Tat variants involving the second exon. This could indicate that both exons of Tat are necessary to have stable folding. The first NMR study on Tat was also carried out with an inactive form of Tat due to the reducing conditions used, but long-range NMR constraints were identified with this protein that had both exons [26].

Previous studies have examined the effect of Zn^2+ ^binding on the structure of Tat with different results [20-22]. We observed no change in the CD spectra in the presence of Zn^2+ ^confirming the results by Frankel et al. [20] that Zn^2+ ^does not affect Tat folding. However, we proffer no evidence that supports the metal-linked dimer form of Tat. Furthermore, monomeric forms of Tat variants are recognized by antibodies from HIV-1-infected patients [27,28].

The C terminus of Tat Eli is packed between the basic region and the cysteine-rich region (Figure 6). The second exon of Tat is composed of three β-turns and is well exposed to solvent. Conformational epitopes exist in Tat variants that influence the magnitude and breadth of antibody response against Tat [10]. These mutations do not prevent the biological activity but dramatically change its immunogenic properties [10]. For instance, antibodies raised against Tat Eli have weak avidity against other Tat variants [10]. Interestingly, a Tat variant called Oyi identified in patients who did not progress to AIDS has a 3D epitope that raised antibodies capable of recognizing all Tat variants. Therefore, the humoral immune response against different Tat variants suggests, as our NMR studies suggest, that a conserved folding exists among Tat variants [10].

Tat Eli has fewer long-range NMR constraints compared to Tat Bru (Figure 5A) and Tat Mal [14,15]. It is possible that some long-range NMR constraints were not detected due to chemical shift overlaps such as for the rings of Trp 11 and Phe 38 (additional file 1). However, Tat Eli has greater trans-activational activity than both Tat Bru and Tat Mal [12], which could be due to greater flexibility compared with these two Tat proteins. This may explain the lower number of long-range NMR constraints.

The exact mechanism by which Tat enters cells remains unknown. The high flexibility and high activity of Tat Eli make it a good candidate to study this mechanism. The core of Tat Eli is mainly composed of 10 aromatic residues organized in a hydrophobic cluster. This core region might be involved during Tat internalization, as the mechanism certainly requires a structural change for this hydrophobic environment. Therefore, it might be interesting to study the structure of Tat Eli or fragments of this protein using solid-state NMR [26] in a hydrophobic environment similar to biological membranes. This, however, is still an ambitious task as it will require uniform (or extensive) ^13^C, ^15^N-labelling and thereby the establishment of appropriate systems for large-scale recombinant expression.

## Conclusion

In conclusion, this study suggests that biologically active Tat variants share a common folding. This study should help to understand how some antibodies neutralize Tat activity and aid the development of an AIDS vaccine targeting Tat. Tat Eli is one of the most active Tat variants that we have synthesized but this variant does not have the capacity to induce a broad immune response against other Tat variants as Tat Oyi does. Therefore, it would be interesting to determine the NMR structure of Tat Oyi (101 residues) and compare it with Tat Eli. Finally, this NMR study of Tat Eli in solution constitutes the basis for a future study that will determine the structural changes required for Tat to traverse cellular membranes using solid-state NMR.

## Methods

### Protein synthesis, purification and characterization

The primary sequence of Tat Eli is MDPVDPNLEPWNHPGSQPRTPCNKCHCKKCCYHCPVCFLNKGLGISYGRKKRRQRRGPPQGGQAHQVPIPKQPSSQPRGDPTGPKEQKKKVESEAETDP. Tat Eli was synthesized in solid phase using Fast Fmoc (9-fluoenylmethoxy carbonyl) chemistry by the method of Barany and Merrifield [29] using 4-hydroxymethyl-phenoxymethyl-copolystyrene-1% divinylbenzene preloaded resin (0.65 mmol) (Perkin Elmer) on an automated synthesizer (ABI 433A, Perkin Elmer) as previously described [12]. Purification was carried out using a Beckman high-pressure liquid chromatography (HPLC) apparatus with a Beckman C8 reverse phase column (10 × 150 mm). Buffer A was water supplemented with 0.1% (v/v) trifluoroacetic acid (Sigma) and buffer B was acetonitrile (Merck) supplemented with 0.1% (v/v) trifluoroacetic acid. Gradient was buffer B from 15–35% in 40 minutes with a 2 ml/min flow rate. HPLC analysis was carried out using a Merck Chromolith™ Performance RP-8e (4.6 × 100 mm) with similar buffers but using a gradient from 10–50% in 15 minutes with a 1.8 ml/min flow rate. Purity of the protein was up to 95%. Amino-acid analyses were performed on a model 6300 Beckman analyzer and mass spectrometry was carried out using an Ettan matrix-assisted laser desorption ionization time-of-flight apparatus (Amersham Biosciences). The synthetic Tat HXB2(86) was previously described [6]. Recombinant Tat was obtained through the NIH AIDS Research and Reference Reagent Program, Division of AIDS, NIAID, NIH from Dr. John Brady and DAIDS, NIAID [30].

### Trans-activation assay with HIV-1 long terminal repeat transfected HeLa cells

The *trans*-activation activities of the synthetic Tat proteins were analyzed by monitoring the production of β-galactosidase after activation of *lacZ *expression in HeLa-P4 cells [31] using a previously described protocol [6,10]. Briefly, 2 × 10^5 ^cells per well were incubated in 24-well flat-bottomed plates (Falcon) at 37°C, 5% CO_2_, in Dulbecco's Modified Eagles Medium (DMEM) supplemented with 10% (v/v) heat-inactivated fetal bovine serum and 100 μg/ml neomycin (all Invitrogen) After 24 h, cells were washed with phosphate-buffered saline. Tat protein was directly mixed with DMEM supplemented with 0.01% (w/v) protamine (Sigma) and 0.1% (w/v) bovine serum albumin (BSA; Sigma) and added to the cells. After 16 hours at 37°C, 5% CO_2_, cells were washed with phosphate-buffered saline, lysed and the β-galactosidase content was measured with a commercially available antigen capture enzyme-linked immunosorbent assay (β-galactosidase ELISA, Roche Diagnostics). Results were normalized using the Bradford reagent (Sigma). Control corresponds to the background β-galactosidase expressed by HeLa-P4 cells in DMEM supplemented with 0.01% (w/v) protamine and 0.1% (w/v) BSA with vehicle and without Tat. Concentrations of Tat used are noted in the figure legend.

### Circular Dichroism

CD spectra were measured with a 100 μm path length from 260–178 nm at 10–70°C on a JASCO J-810 spectropolarimeter. Data were collected at 0.5 nm intervals using a step auto response procedure (JASCO). CD spectra are presented as Δε per amide. Protein concentration was 1 mg/ml in 20 mM pH 4.5 phosphate buffer for the three proteins: BSA, protamine, and Tat Eli and in 20 mM pH 7 phosphate buffer for Tat Eli with 0 to 16 molar equivalents of ZnCl_2_. The CD data were analyzed with VARSELEC to determine the secondary structure content according to the method of Manavalan and Johnson [32] using a set of 32 reference proteins and an average of 4960 calculations.

### NMR spectroscopy

Tat samples for NMR (1 mM) were prepared in H_2_O/D_2_O [9:1] 100 mM phosphate buffer at pH 4.5. The homonuclear ^1^H NMR spectra were recorded on a Varian Inova 800 MHz NMR spectrometer operating at 799.753 MHz. ^1^H TOCSY spectra [33,34] with 80 ms mixing, and NOESY spectra [35] with 50, 100, 150, and 200 ms mixing, were recorded at 20°C with a spectral width of 10999.588 Hz. The water signal was suppressed using weak presaturation (2 s). Data were processed with the Felix 2002 from Accelrys (San Diego, CA).

### Molecular modeling

Molecular modeling was performed using the Insight II 2002 package including Biopolymer, Discover, Homology and NMR-refine software (Accelrys, San Diego, CA). High temperature simulated annealing was carried out according to Nilges et al. [23].

## Competing interests

The authors declare that they have no competing interests.

## Authors' contributions

JDW carried out the trans-activation assays, circular dichroism and NMR studies and was involved in drafting and revising the manuscript, GRC helped synthesize Tat proteins, participated in the design of the study, carried out trans-activation assays and was involved in drafting and revising the manuscript, HH participated in the CD assays, EPL conceived of the study, participated in its design, coordination, analysis and interpretation of data and drafted the manuscript. All authors read and approved the final manuscript.

## Supplementary Material

Additional file 1 Table I: 1H Chemical Shifts of Tat Eli at 293 K in Phosphate Buffer (pH 4.5). Click here for file

Additional file 2 TABLE II. Structural statistics and Root Mean Square Deviation (RMSD) for 8 conformers obtained from Simulated Annealing (SA) and final structure obtained from energy minimization of mean structure. Click here for file
