# Supplementary material for: Homonuclear 1H NMR and circular dichroism study of the HIV-1 Tat Eli variant
Source: Retrovirology. 2008 Sep 22;5:83. doi: 10.1186/1742-4690-5-83 (PMC2557015; doi:10.1186/1742-4690-5-83)
Supplement: Additional file 1 — Table I: 1H Chemical Shifts of Tat Eli at 293 K in Phosphate Buffer (pH 4.5). [file 1742-4690-5-83-S1.pdf]

TABLE I ^{1}H Chemical Shifts of Tat Eli at 293K in Phosphate Buffer (pH 4.5)

**Résidus**	**NH**	**H**a	**H**b	**H**g	**Others**
METn_ 1		4.030	2.071	2.503	
ASP-_ 2	8.895	4.905	2.814/2.594		
PRO_3		4.270	2.164	1.936/1.896	δCH2 3.759
VAL_4	8.095	3.928	1.920	0.845/0.801	
ASP-_5	8.399	4.845	2.840/2.644		
PRO_6		4.300	2.164	1.936/1.895	δCH2 3.758
ASN-7	8.187	4.523	2.728/2.633		δ 7.593/6.879
LEU_8	7.786	4.198	1.535/1.535	1.440	δCH3 0.823/0.732
GLU-_9	7.942	4.225	1.458/1.180	2.240	
PRO_10		4.188	2.140	1.881/1.788	δCH2 3.499/3.368
TRP_11	7.317	4.549	3.228/3.212		2H 7.126; 4H 7.092; 5H 7.159; 6H 7.388; 7H 7.532; NH 10.167
ASN_12	7.664	4.575	2.452/2.282		δ 7.312/6.746
HIS+_13	7.901	4.747	3.061/2.935		2H 8.474; 4H 7.054
PRO_14		4.357	2.205	1.936/1.896	δCH2 3.589/3.472
GLY_15	8.589	3.965/3.897			
SER_16	8.119	4.353	3.770		
GLN_17	8.368	4.515	1.986/1.827	2.283	δCH2 7.500/6.900
PRO_18		4.366	2.191	1.936/1.815	δCH2 3.670
ARG+_19	8.472	4.305	1.781/1.687	1.595/1.559	δCH2 3.118
THR_20	8.161	4.490	4.046	1.155	
PRO_21		4.330	2.222	1.953/1.899	δCH2 3.772/3.616
CYSH_22	8.462	4.380	2.836		
ASN_23	8.499	4.614	2.728		δ 7.500/6.800
LYS+_24	8.495	4.232	1.750/1.674	1.385/1.339	δCH2 1.599; εCH2 2.900; εNH3+ 7.470
CYSH_25	8.217	4.313	2.797		
HIS+_26	8.620	4.663	3.210/3.100		2H 8.503; 4H 7.124
CYSH_27	8.276	4.423	2.835		
LYS+_28	8.283	4.181	1.710/1.651	1.367/1.308	δCH2 1.588; εCH2 2.906; εNH3+ 7.467
LYS+_29	8.339	4.180	1.711/1.659	1.362/1.312	δCH2 1.600; εCH2 2.907; εNH3+ 7.476
CYSH_30	8.405	4.884	3.194		
CYSH_31	8.402	4.394	2.768		
TYR_32	8.211	4.434	2.830/2.796		2H,6H 6.981/6.794; 3H,5H 6.706/6.698
HIS+_33	8.207	4.510	3.052/2.945		2H 8.500; 4H 7.136
CYSH_34	8.370	4.535	2.796		
PRO_35		4.408	2.218	1.930/1.828	δCH2 3.721
VAL_36	8.165	3.919	1.900	0.837/0.746	
CYSH_37	8.241	4.375	2.755/2.728		
PHE_38	8.278	4.565	3.083/2.894		2H, 6H 7.154; 3H, 5H 7.253/7.203
LEU_39	8.034	4.205	1.502	1.447	δCH3 0.810/0.762
ASN_40	8.312	4.546	2.760/2.662		δ 7.563/6.870
LYS+_41	8.241	4.190	1.783	1.360/1.306	δCH2 1.546; εCH2 2.889; εNH3+ 7.467
GLY_42	8.329	3.843			
LEU_43	8.019	4.221	1.565	1.506	δCH3 0.807/0.760
GLY_44	8.358	3.843			
ILE_45	7.882	4.044	1.691	1.300/1.020	γCH3 0.740/0.686
SER_46	8.267	4.349	3.711		
TYR_47	8.143	4.444	2.995/2.847		2H, 6H 7.028/7.020; 3H, 5H 6.729/6.720
GLY_48	8.278	3.803			
ARG+_49	8.076	4.204	1.761/1.663	1.546/1.509	δCH2 3.104
LYS+_50	8.401	4.176	1.735/1.681	1.389/1.342	δCH2 1.595; εCH2 2.916; εNH3+ 7.476
LYS+_51	8.360	4.212	1.724/1.669	1.361/1.295	δCH2 1.585/1.513; εCH2 2.905; εNH3+ 7.467
ARG+_52	8.448	4.215	1.734/1.670	1.568/1.518	δCH2 3.102
ARG+_53	7.847	4.213	1.752	1.568	δCH2 3.118
GLN_54	8.395	4.269	1.938/1.847	2.231	
ARG+_55	8.472	4.330	1.774/1.692	1.559	δCH2 3.118
ARG+_56	8.031	4.383	1.795	1.559	δCH2 3.104
GLY_57	8.286	4.089/3.928			
PRO_58		4.632	2.251	1.909/1.840	δCH2 3.556/3.512
PRO_59		4.340	2.195	1.926/1.819	δCH2 3.712
GLN_60	8.513	4.238	2.030/1.904	2.308	δCH2 7.503/6.835
GLY_61	8.435	3.902			
GLY_62	8.277	3.872			
GLN_63	8.247	4.231	1.993/1.855	2.256	δCH2 7.503/6.835
ALA_64	8.292	4.152	1.231		
HIS+_65	8.415	4.595	3.169/3.064		2H 8.500; 4H 7.203

**Résidus**	**NH**	**Ha**	**Hb**	**Hg**	**Others**
GLN_66	8.395	4.529	2.004/1.847	2.315	δCH2 7.505/6.837
VAL_67	8.262	4.326	1.994	0.902/0.858	
PRO_68		4.326	2.171/1.944	1.894/1.748	δCH2 3.805/3.593
ILE_69	8.208	4.330	1.747	1.474/1.115	γCH3 0.876/0.786
PRO_70		4.304	2.203	1.909/1.780	δCH2 3.590
LYS+_71	8.294	4.181	1.702/1.648	1.368/1.309	δCH2 1.591; εCH2 2.898; εNH3+ 7.470
GLN_72					
PRO_73		4.425	2.220	2.245	δCH2 3.725
SER_74	8.439	4.367	3.833/3.788		
SER_75	8.289	4.392	3.780		
GLN_76	8.239	4.259	1.989/1.882	2.345	
PRO_77		4.425	2.220	1.947/1.823	δCH2 3.725/3.686
ARG+_78	8.084	4.207	1.765/1.667	1.557/1.523	δCH2 3.106
GLY_79	8.519	3.887/3.802			
ASP-_80	8.328	4.888	2.792/2.600		
PRO_81		4.306	2.204	1.909/1.775	δCH2 3.820
THR_82	8.110	4.290	4.198	1.133	
GLY_83	8.065	4.076/3.987			
PRO_84		4.340	2.221	1.926/1.819	δCH2 3.551
LYS+_85	8.247	4.219	1.792	1.358/1.303	δCH2 1.672/1.575; εCH2 2.906; εNH3+ 7.473
GLU-_86	8.247	4.231	1.993/1.861	2.256	
GLN_87	8.470	4.235	1.971/1.887	2.272	
LYS+_88	8.265	4.222	1.649	1.357/1.308	δCH2 1.581; εCH2 2.909; εNH3+ 7.470
LYS+_89					
LYS+_90					
VAL_91	8.186	4.010	1.961	0.847	
GLU-_92	8.446	4.330	2.030/1.833	2.378	
SER_93	7.965	3.943	3.675		
GLU-_94	8.277	4.558	1.996/1.842	2.304	
ALA_95	8.384	3.145	1.314		
GLU-_96	8.303	4.325	2.051/1.896	2.390	
THR_97	8.636	4.333	4.123	1.167	
ASP-_98	8.300	4.802	2.800/2.700		
PRO_99		4.440	2.205	1.925	δCH2 3.776/3.722
