# Supplementary material for: Homonuclear 1H NMR and circular dichroism study of the HIV-1 Tat Eli variant
Source: Retrovirology. 2008 Sep 22;5:83. doi: 10.1186/1742-4690-5-83 (PMC2557015; doi:10.1186/1742-4690-5-83)
Supplement: Additional file 2 — TABLE II. Structural statistics and Root Mean Square Deviation (RMSD) for 8 conformers obtained from Simulated Annealing (SA) and final structure obtained from energy minimization of mean structure. [file 1742-4690-5-83-S2.pdf]

TABLE II. Structural statistics and Root Mean Square Deviation (RMSD) for 8 conformers obtained from Simulated Annealing (SA) and final structure obtained from energy minimization of mean structure

	Average for SA Structures	Final Structure
Agreement with 1639 NMR	1474	1474
RMSD from idealized geometry		
Bonds (Å)	0,036	0,028
Angles (°)	7,5	4,8
Quality of Ramachandran map [a]		
Residues in		
Most favored regions	32,3	38,4
Additional allowed regions	33,8	27,4
Generously allowed regions	21,7	20,5
Disallowed regions	12,2	13,7
van der Waals [b]	2825	372
Electrostatic [b]	940,9	620,1

[a] Data obtained from a Procheck validation procedure

[b] Energies (kcal.mol-1) determined from Accelrys, Insight II software.
